# Mechanisms of Chromium and Uranium Toxicity in *Pseudomonas stutzeri* RCH2 Grown under Anaerobic Nitrate-Reducing Conditions

**DOI:** 10.3389/fmicb.2017.01529

**Published:** 2017-08-10

**Authors:** Michael P. Thorgersen, W. Andrew Lancaster, Xiaoxuan Ge, Grant M. Zane, Kelly M. Wetmore, Brian J. Vaccaro, Farris L. Poole, Adam D. Younkin, Adam M. Deutschbauer, Adam P. Arkin, Judy D. Wall, Michael W. W. Adams

**Affiliations:** ^1^Department of Biochemistry and Molecular Biology, University of Georgia Athens, GA, United States; ^2^Department of Biochemistry, University of Missouri Columbia, MO, United States; ^3^Environmental Genomics and Systems Biology Division, Lawrence Berkeley National Laboratory Berkeley, CA, United States

**Keywords:** anaerobes, nitrate reductase, transposon mutagenesis, metals, heavy, contaminated groundwater

## Abstract

Chromium and uranium are highly toxic metals that contaminate many natural environments. We investigated their mechanisms of toxicity under anaerobic conditions using nitrate-reducing *Pseudomonas stutzeri* RCH2, which was originally isolated from a chromium-contaminated aquifer. A random barcode transposon site sequencing library of RCH2 was grown in the presence of the chromate oxyanion (Cr[VI]${\text{O}}_{4}^{2-}$) or uranyl oxycation (U[VI]${\text{O}}_{2}^{2+}$). Strains lacking genes required for a functional nitrate reductase had decreased fitness as both metals interacted with heme-containing enzymes required for the later steps in the denitrification pathway after nitrate is reduced to nitrite. Cr[VI]-resistance also required genes in the homologous recombination and nucleotide excision DNA repair pathways, showing that DNA is a target of Cr[VI] even under anaerobic conditions. The reduced thiol pool was also identified as a target of Cr[VI] toxicity and *psest_2088*, a gene of previously unknown function, was shown to have a role in the reduction of sulfite to sulfide. U[VI] resistance mechanisms involved exopolysaccharide synthesis and the universal stress protein UspA. As the first genome-wide fitness analysis of Cr[VI] and U[VI] toxicity under anaerobic conditions, this study provides new insight into the impact of Cr[VI] and U[VI] on an environmental isolate from a chromium contaminated site, as well as into the role of a ubiquitous protein, Psest_2088.

## Introduction

The industrial use of chromium for metallic plating, industrial catalysts, and pesticides has led to wide scale environmental contamination (Ayres, 1992). For example, there are high levels of Cr contamination at the Hanford 100-Area in Washington State, a 26-square-mile area along the Columbia River, where several water-cooled plutonium reactors were constructed and operated (Fruchter, 2002). Environmental chromium is problematic since exposure to the element poses significant risks to human health causing skin ulcers, respiratory ailments, allergic reactions and cancer (Grevatt, 1998; Dayan and Paine, 2001). Due to its high toxicity, chromium is considered a priority pollutant and the maximum amount of chromium allowed in drinking water by the US Environmental Protection Agency is 0.1 mg/L (Cheung and Gu, 2007; Gupta et al., 2011).

Chromium exists in several different oxidation states from −4 to +6 with the oxyanion hexavalent state (Cr[VI]) and the oxyanion trivalent state (Cr[III]) being the most stable (Cervantes et al., 2001; Cheung and Gu, 2007). Of these two oxidation states, Cr[VI] is over 1,000-fold more toxic as it is highly soluble and can be transported across membranes via sulfate transport channels (Ohtake and Silver, 1994; Cervantes et al., 2001; Costa, 2003). In contrast, Cr[III] species are largely impermeable (Czakó-Vér et al., 1999). Once inside the cell, there are several ways in which Cr causes toxicity. Cr[VI] is reduced to Cr[V] and Cr[III] by compounds such as glutathione and ascorbic acid, a process that also generates reactive oxygen species (ROS) (Arslan et al., 1987; Costa, 2003; Xu et al., 2004). These intracellular reduced Cr species have additional toxic effects, including Cr[III] binding to cellular proteins and DNA, and the formation of ROS by the reoxidation of Cr[V] (Kortenkamp and O'brien, 1994; Costa, 2003). Cr[VI] toxicity is known to be both mutagenic and carcinogenic causing both frameshift and basepair substitution mutations (Venitt and Levy, 1974; Nishioka, 1975; Petrilli and De Flora, 1977).

Chromate resistance mechanisms involving efflux have been characterized from several different microorganisms (Ramírez-Díaz et al., 2008; Thatoi et al., 2014). The efflux protein ChrA is encoded on plasmids in *Pseudomonas aeruginosa* and *Cupriavidus metallidurans* (Cervantes-Cervantes et al., 1990; Nies et al., 1990; Alvarez et al., 1999) and transports Cr[VI] to outside of the cell membrane using proton motive force (Alvarez et al., 1999; Pimentel et al., 2002). Other microorganisms respond to Cr exposure by inducing the expression of genes that combat oxidative stress as a defense mechanism. For example, *Escherichia coli* increases production of ROS detoxification enzymes such as superoxide dismutase (SOD) and catalase (Ackerley et al., 2006) and *Shewanella oneidensis* MR-1 generates increased concentrations of thioredoxins and glutaredoxins (Chourey et al., 2006). DNA repair systems are also induced in response to aerobic chromate exposure, including components of the DNA SOS repair system and the Rec system (Ramírez-Díaz et al., 2008).

Uranium is a highly toxic industrial element that contaminates natural environments through processes such as mining and milling (Beneš, 1999). The U.S. Department of Energy (DOE) oversees the monitoring and restoration of uranium contamination at 12 facilities nationwide (Riley and Zachara, 1992). In groundwater, uranium is usually present in either the U[VI] or U[IV] oxidation states. Previous studies have shown that microorganisms can sequester or precipitate uranium extracellularly in several different ways (Marqués et al., 1990; Lovley et al., 1993; Merroun et al., 2003, 2005, 2011; Martins et al., 2010; Thorgersen et al., 2016), but it is unclear if these U immobilization methods act as defense mechanisms for the microorganisms involved.

Herein we report the investigation of chromium and uranium toxicity on a denitrifying bacterium growing under anaerobic conditions. *Pseudomonas stutzeri* RCH2 (RCH2) was isolated from a Cr-contaminated aquifer at the Hanford 100H site (Han et al., 2010). A random barcode transposon site sequencing (RB-TnSeq) library was created for RCH2 allowing for convenient gene function analysis on a genome wide scale (Wetmore et al., 2015), and this library has been used to determine gene fitness under a number of metal-related conditions, including Mo limitation (Vaccaro et al., 2016b) and Cu/Zn toxicity (Vaccaro et al., 2016a). Herein we grew the RCH2 RB-TnSeq librry under conditions of Cr[VI] and U[VI] stress to determine the main toxicity targets of these metals in RCH2 under denitrifying conditions, and to determine key defense mechanisms RCH2 has against these metals. The resulting fitness data provide new insights into the effects of Cr[VI] and U[VI] on the denitrification pathway, which could impact remediation in sites contaminated with both heavy metals and nitrate. The data also led to the characterization of hypothetical gene *psest_2088* in RCH2, which is involved in sulfur assimilation. While there have been many studies on the toxic effects of Cr[VI] and U[VI] using microorganisms grown under aerobic conditions, this is the first in depth look at Cr[VI] and U[VI] toxicity in an anaerobic denitrifying system on a genome wide scale.

## Materials and methods

### Growth conditions

The basal growth medium had the following composition: 20 mM sodium fumarate, 20 mM NaNO_3_, 4.7 mM NH_4_Cl, 1.3 mM KCl, 2 mM MgSO_4_, 0.2 mM NaCl, 1.2 mM NaHCO_3_, 5 mM NaH_2_PO_4_, 0.1 mM CaCl_2_ with sterile vitamins and trace elements prepared as described by Widdel and Bak (1992). Initial cultures were grown aerobically. These were then diluted 20-fold into the experimental growth medium. For each experiment, where applicable, the indicated amounts of exogenous sulfur sources, uranyl acetate (U[VI]) and K_2_Cr_2_O_7_ (Cr[VI]) were added to the basal growth medium. Cultures were grown in a 100 well Bioscreen plate with each well containing 400 μL of diluted preculture. The Bioscreen plate was incubated anaerobically or aerobically as indicated at 30°C with continuous shaking in a Bioscreen C (Thermo Labsystems, Milford, MA). In the case of anaerobic growths, the Bioscreen C was placed within an anaerobic chamber (Plas Labs, Lansing, MI) in an atmospheric composition of 95% Ar and 5% H_2_. Growth was monitored at an absorbance of 600 nm. All experiments were performed in biological duplicate or triplicate, errors bars represent standard deviations.

### Mutant library growth

The *P. stutzeri* RCH2 RB-TnSeq mutant library containing 166,448 single transposon mutations with mapped genome locations (Wetmore et al., 2015) was recovered from a 1 mL, 10% glycerol stock at −80°C by incubating aerobically with shaking (150 rpm) at 30°C for 5.5 h in 125 mL of Luria broth with 50 μg/mL kanamycin in a shake flask to an OD_600_ of 1.0. A sample of the recovery culture was saved as the pregrowth condition. The fitness growths were carried out in triplicate in the basal growth medium described above except 20 mM sodium lactate was used as a carbon source instead of fumarate, and 0.5 g/L yeast extract were added. No additional metals beyond the trace metals solution were added to the control cultures, while 120 μM K_2_Cr_2_O_7_ (240 μM Cr[VI]) was added to the Cr challenge cultures and 3 mM uranyl acetate (U[VI]) was added to the U challenge cultures. The Cr and U concentrations were chosen as the concentrations that decreased growth (OD_600_) under the fitness growth conditions approximately 50%. Cultures (5 mL) in sealed anaerobic Hungate tubes with an argon atmosphere were inoculated to an OD_600_ of 0.02 before incubating with shaking (150 rpm) at 30°C for 5 h. At the end of growth, the OD_600_ of each culture was recorded and the cultures were saved as postgrowth samples.

### DNA isolation, PCR, sequencing, and sequence analysis

The processing of the cultures for DNA isolation, DNA sequencing, and sequence analysis were carried out as previously described with the BarSeq98 method (Wetmore et al., 2015). The Illumina HiSeq system was used to sequence PCR products. Strain fitness defined as the binary logarithm of the ratio of postgrowth to pregrowth relative abundances were calculated for each individual transposon insertion strain. Gene fitness values (*w*) were calculated as previously described (Wetmore et al., 2015), by averaging the fitness values for strains with insertions in a given gene. Quality control and normalization of data were performed as previously reported (Wetmore et al., 2015; Vaccaro et al., 2016a). The quality of each experiment was evaluated using several criteria. Including, the number of counts for the median gene needed to be greater than or equal to 50, and the median absolute difference in fitness between the two havles of the genes (mad12) was less than or equal to 0.5. Quality metrics for the fitness data are reported for each growth condition in Table S1.

### Generation of deletion mutant strain Δ2088

The marker-exchange deletion strain, Δ2088, used in this study was constructed by conjugation of an unstable, marker-exchange plasmid into RCH2 with selection for Kan^r^, in a manner previously described (Vaccaro et al., 2016a). The plasmid was made with the primers found in Table S2.

### Reduced intracellular thiol assay

Cultures (300 mL) of *P. stutzeri* RCH2 wild-type and Δ2088 were grown in triplicate anaerobically on basal medium in sealed bottles at 30°C with or without 50 μM K_2_Cr_2_O_7_ as indicated to late log phase. The cultures were harvested by centrifugation for 10 min at 4°C and 7,500 RPM. Cell pellets were moved into an anaerobic chamber (Coy Laboratory Products, Grass Lake, MI), with an atmospheric composition of 95% Ar and 5% H2, where they were washed once with 50 mM Tris pH 8.0, and suspended in the same buffer at a volume of 3 mL to 1 g of pellet (wet weight). Lysozyme (0.1 mg/mL) and deoxy ribonuclease (0.1 mg/mL) were added to the cell suspensions, which were then lysed by sonication and centrifuged at 10,000 RPM for 10 min. The supernatant was saved as cell free extract, and the Bradford assay was used to quantitate protein concentration (Bradford, 1976).

Total free thiol concentrations were measured from the cell free extracts using the Ellman assay (Sedlak and Lindsay, 1968). Briefly, 20 μL of cell free extract was combined with 75 μL of 30 mM Tris, 3 mM EDTA pH 8.2; 25 μL of 150 μM DTNB dissolved in methanol; and 400 μL of methanol. Samples were spun at 7.5 rpm for 5 min and 270 μL were transferred to a microplate for absorption measurement at 412 nm. A standard curve of reduced glutathione dissolved in 20 mM triethanolamine-HCl was used to convert absorbance values to moles of reduced thiol groups/g protein. Error is reported as the standard deviation between biological triplicates.

### Whole cell nitrite reductase assays

RCH2 cultures (5 mL) were grown anaerobically in crimp sealed Hungate tubes under a 100% argon atmosphere with shaking (150 rpm) at 30°C for 5 h. Cultures in triplicate contained either no additional metal, 3 mM uranyl acetate, or 120 μM K_2_Cr_2_O_7_. Preparation of whole cells and nitrite reductase assays were performed as previously described (Thorgersen and Adams, 2016). Nitrite reductase values are reported as Units/mg protein, where a unit corresponds to 1 nmol of nitrite reduced/min.

## Results/discussion

### Experimental approach and analysis of RB-TnSeq data

Fitness experiments were conducted using the previously described RCH2 RB-TnSeq mutant library containing 166,448 single transposon mutations with mapped genome locations (Wetmore et al., 2015). The library was grown anaerobically using fumarate (20 mM) as the carbon source under denitrifying conditions with 20 mM nitrate in the presence of either no additional metal (control), 120 μM K_2_Cr_2_O_7_ (Cr[VI]) or 3 mM uranyl acetate (U[VI]). Metal concentrations were selected that would inhibit growth of RCH2 by approximately 50%. Gene fitness values (*w*) are a measure of the population of mutants with disruptions in an individual gene relative to the overall mutant library population and these were calculated for each gene under all growth conditions as previously described (Wetmore et al., 2015). An increase in the relative abundance of mutants in a given gene in the test condition compaired to the pregrowth condition results in a positive fitness value and a decrease results in a negative fitness value. Both the Cr[VI] and U[VI] challenge growth gene fitness values were compared to the control grown in the absence of these metals to determine genes whose fitness was increased or decreased as a result of the metal challenge. Gene fitness values for the Cr[VI] (*w*_Cr_) and U[VI] (*w*_U_) challenges that have been corrected by the control gene fitness values (*w*_Cont_) will be referred to by the symbols Δ*w*_Cr_ and Δ*w*_U_. Genes with Δ*w*_Cr_ and Δ*w*_U_ ≤ −1.0 are listed in Tables 1, 2 respectively. Genes with Δ*w*_Cr_ and Δ*w*_U_ values ≥1.0 are listed in Tables S3, S4 respectively. Analysis of the potential influence of polar effects in the RCH2 mutant library was previously conducted (Wetmore et al., 2015), and it was conluded that they do not have a major effect. The false discovery rate for genes with phenotypes was also estimated for the library, and the rate was under 2% (Wetmore et al., 2015).

**Table 1:** Genes with Δ*wCr* ≤ −1.

**Locus Tag**	**Gene Function**	***w_*ctrl*_***	***w_*ctrl*_***	***wCr***	***wCr***	**Δ*wCr***
		**AVE**	**STDEV**	**AVE**	**STDEV**	**Delta**
**DNA REPAIR**						
Psest_3090	DNA repair protein, RecO	−0.2	0.1	−2.1	0.4	−1.9
Psest_0004	DNA repair protein, RecF	−0.2	0.1	−2.1	0.1	−1.9
Psest_2545	Recombination protein, RecR	−0.2	0.3	−2.0	0.5	−1.8
Psest_2646	SOS regulatory protein, LexA repressor	−0.5	0.4	−2.2	1.0	−1.7
Psest_0212	Exodeoxyribonuclease V, RecC	0.6	0.8	−1.0	0.5	−1.6
Psest_2259	Excinuclease ABC, UvrC	0.2	0.1	−1.2	0.1	−1.4
Psest_2872	DNA repair protein, RecA	−0.7	0.8	−2.0	0.4	−1.3
Psest_2647	SOS-response cell division inhibitor, SulA	−0.2	0.1	−1.6	0.2	−1.3
**SULFUR ASSIMILATION**						
Psest_4316	ABC-type Methionine transport system	−0.7	0.3	−3.7	0.4	−3.0
Psest_2088	Uncharacterized protein conserved in bacteria	−0.4	1.3	−2.7	0.7	−2.3
Psest_0494	Rhodanese-related sulfurtransferase	−0.2	0.0	−2.4	0.1	−2.1
Psest_4314	ABC-type methionine transport system, MetN	−0.6	0.1	−2.6	0.3	−2.0
Psest_4315	ABC-type Methionine transport system	−0.8	0.5	−2.3	0.4	−1.5
Psest_4063	Sulfate ABC transporter	0.2	0.2	−0.8	0.2	−1.0
**NITRATE REDUCTION AND MO COFACTOR BIOSYNTHESIS**						
Psest_3482	Parvulin-like peptidyl-prolyl isomerase	−0.3	0.3	−2.6	0.5	−2.3
Psest_0811	Heme d1 biosynthesis radical SAM protein, NirJ	−0.5	0.1	−2.2	0.2	−1.7
Psest_3481	MoCo biosynthesis protein A, MoaA	−0.4	0.3	−1.9	0.1	−1.5
Psest_3480	MoCo biosynthesis protein B, MoaB	−1.6	0.6	−3.0	1.6	−1.4
Psest_3479	MoCo synthesis domain, MoeA	−2.1	0.3	−3.4	0.1	−1.3
Psest_3000	Molybdate ABC transporter	−1.2	0.2	−2.3	0.2	−1.2
Psest_3486	Respiratory nitrate reductase, NarG	−1.8	0.1	−2.9	0.2	−1.1
Psest_3485	Nitrate reductase, NarH	−2.1	0.1	−3.1	0.1	−1.0
**HYPOTHETICAL**						
Psest_2557	Protein of unknown function (DUF2474)	0.0	0.6	−1.6	0.7	−1.6
Psest_3230	Uncharacterized conserved protein	−0.2	0.1	−1.6	0.5	−1.4
Psest_0820	Hypothetical protein	−0.7	0.1	−2.1	0.4	−1.4
Psest_2090	Protein of unknown function (DUF2970)	−0.4	0.2	−1.7	0.2	−1.3
Psest_2756	Hypothetical protein	0.5	0.4	−0.7	0.4	−1.2
Psest_2026	Uncharacterized conserved protein	−0.4	0.2	−1.5	0.2	−1.2
Psest_1920	Uncharacterized conserved protein	−0.9	0.5	−2.1	0.4	−1.2
Psest_2324	Hypothetical protein	−0.9	0.2	−2.1	0.1	−1.1
Psest_2561	Hypothetical protein	−0.2	0.3	−1.2	0.7	−1.1
Psest_1563	Protein of unknown function (DUF548)	0.0	0.5	−1.0	0.2	−1.0
**OTHER**						
Psest_1957	Outer membrane porin, OprD family.	−0.2	0.1	−3.2	0.4	−3.0
Psest_3301	Predicted transcriptional regulator	−1.8	0.2	−4.2	0.9	−2.4
Psest_3721	Malic enzyme	−0.2	0.1	−1.9	0.4	−1.7
Psest_1231	Na+/H+ antiporter, NhaD	−0.4	0.2	−2.1	0.2	−1.7
Psest_2975	tRNA_Arg_CCT	0.5	0.3	−1.1	0.1	−1.6
Psest_0815	Cytochrome C, NirS	−0.5	0.2	−2.1	0.1	−1.6
Psest_0830	cAMP-binding proteins	−0.8	0.2	−2.4	0.5	−1.5
Psest_0821	Cytochrome D1 heme domain, NirF	−0.6	0.1	−2.1	0.2	−1.5
Psest_1873	Predicted permease, DMT superfamily	0.3	0.4	−1.2	0.3	−1.5
Psest_0817	Ethylbenzene dehydrogenase.	−0.7	0.1	−2.1	0.4	−1.5
Psest_0823	Transcriptional regulators	−0.6	0.4	−2.1	0.2	−1.5
Psest_0449	Glutamine synthetase adenylyltransferase	−0.2	0.1	−1.7	0.1	−1.4
Psest_0855	NAD-dependent aldehyde dehydrogenases	−0.8	0.2	−2.2	0.1	−1.4
Psest_2325	alpha-L-glutamate ligase-related protein	−0.6	0.3	−2.0	0.4	−1.4
Psest_2285	ATP-dependent protease La	0.2	1.0	−1.1	0.2	−1.4
Psest_2027	ATP-dependent Clp protease, ClpA	0.0	0.1	−1.4	0.2	−1.3
Psest_3731	Exopolyphosphatase	−0.4	0.1	−1.7	0.0	−1.3
Psest_1824	Sugar transferase	0.2	1.5	−1.1	0.1	−1.2
Psest_0956	Transcriptional regulators	0.1	0.2	−1.1	0.1	−1.2
Psest_1888	Sua5/YciO/YrdC/YwlC family protein	1.4	0.8	0.3	0.7	−1.1
Psest_1640	(p)ppGpp synthetase, RelA/SpoT family	−0.2	0.1	−1.3	0.0	−1.1
Psest_0822	Transcriptional regulators	−0.3	0.6	−1.4	0.2	−1.1
Psest_0759	Protein-L-isoaspartate O-methyltransferase	0.7	0.8	−0.3	0.8	−1.0
Psest_1944	NAD-specific glutamate dehydrogenase	0.0	0.1	−1.0	0.1	−1.0
Psest_1819	Nucleoside-diphosphate-sugar epimerases	−0.6	0.1	−1.5	0.2	−1.0
Psest_0346	Putative solute:sodium symporter small subunit	0.3	0.5	−0.7	0.2	−1.0

**Table 2:** Genes with Δ*wU* ≤ −1.

**Locus Tag**	**Gene Function**	***w_*ctrl*_***	***w_*ctrl*_***	***wU***	***wU***	**Δ*wU***
		**AVE**	**STDEV**	**AVE**	**STDEV**	**Delta**
**NITRATE REDUCTION AND MO COFACTOR BIOSYNTHESIS**						
Psest_3480	MoCo biosynthesis protein B, MoaB	−1.6	0.6	−5.2	1.2	−3.5
Psest_1724	Anti-anti-sigma regulatory factor	−1.5	0.4	−4.2	0.7	−2.7
Psest_0393	Methylase of chemotaxis methyl-accepting proteins	−2.2	0.4	−4.5	0.6	−2.2
Psest_3479	MoCo synthesis domain, MoeA	−2.1	0.3	−4.4	0.1	−2.2
Psest_1115	MoCo biosynthesis, MoeB	−2.6	0.6	−4.6	0.5	−2.0
Psest_3486	Respiratory nitrate reductase, NarG	−1.8	0.1	−3.6	0.2	−1.8
Psest_1961	molybdopterin-guanine dinucleotide biosyn, MobA	−2.4	0.2	−4.1	0.3	−1.7
Psest_3485	Nitrate reductase, NarH	−2.1	0.1	−3.7	0.2	−1.5
Psest_3484	Nitrate reductase MoCo assembly chaperone	−1.8	0.4	−3.3	0.5	−1.5
Psest_3490	Signal transduction histidine kinase, nitrate/nitrite	−1.2	0.9	−2.7	0.6	−1.5
Psest_3483	Respiratory nitrate reductase, NarI	−2.4	0.1	−3.8	0.1	−1.4
Psest_3170	MoCo biosynthesis protein, MoaC	−2.5	0.5	−3.8	0.2	−1.3
Psest_3000	Molybdate ABC transporter	−1.2	0.2	−2.4	0.1	−1.2
Psest_2999	Molybdate ABC transporter	−1.4	0.5	−2.5	0.5	−1.1
**HYPOTHETICAL**						
Psest_3489	Hypothetical protein	−1.3	0.0	−2.9	0.1	−1.5
Psest_3766	Uncharacterized conserved protein	−0.3	1.2	−1.6	0.3	−1.3
Psest_2324	Hypothetical protein	−0.9	0.2	−2.2	0.5	−1.2
Psest_0193	Conserved hypothetical protein	−1.0	0.4	−2.2	0.2	−1.2
Psest_3881	Hypothetical protein	0.3	0.2	−0.7	0.2	−1.0
**OTHER**						
Psest_2232	UTP-glucose-1-phosphate uridylyltransferase	−0.2	0.0	−4.5	0.2	−4.3
Psest_0993	Glucose-6-phosphate isomerase	−0.7	0.1	−3.1	0.3	−2.3
Psest_3488	Universal stress protein, UspA	−1.2	0.4	−3.0	0.1	−1.9
Psest_2325	alpha-L-glutamate ligase-related protein	−0.6	0.3	−2.3	0.2	−1.7
Psest_1805	Integration host factor, IhfB	−0.6	0.8	−2.3	0.7	−1.7
Psest_1888	Sua5/YciO/YrdC/YwlC family protein	1.4	0.8	−0.2	0.4	−1.6
Psest_3960	3′(2′),5′-bisphosphate nucleotidase, bacterial	0.5	0.5	−0.9	0.1	−1.4
Psest_4010	Peroxiredoxin, OsmC subfamily	0.6	1.4	−0.7	0.6	−1.4
Psest_1511	Predicted redox protein	−1.3	0.2	−2.4	0.1	−1.1
Psest_1974	Integration host factor, IhfA	−1.0	0.3	−2.1	0.2	−1.1
Psest_0999	Response regulator	−0.7	0.5	−1.8	0.5	−1.1
Psest_1663	Pyruvate/2-oxoglutarate dehydrogenase complex	−1.3	0.3	−2.4	0.2	−1.1
Psest_1293	VanZ like family	0.1	0.3	−1.0	0.2	−1.0

### Chromate toxicity involving DNA repair and ROS detoxification

In response to exposure to Cr[VI], negative Δ*w*_Cr_ values were observed for a large number of genes involved in DNA repair. These included *recF* (−1.9), *recO* (−1.9), *recR* (−1.8), and *recA* (−1.3), which are all part of the RecFOR pathway involved in homologous recombination to repair single strand breaks (Morimatsu and Kowalczykowski, 2003). The gene *uvrC* involved in nucleotide excision repair also had a large negative Δ*w*_Cr_ (−1.4), as did the genes of the other proteins involved in excision repair, *uvrA* and *uvrB*, that had Δ*w*_Cr_ values of −0.8 each. The gene of a helicase involved in double stranded repair processes, RecC, had a Δ*w*_Cr_ value of −1.6. Interestingly, the gene encoding the SOS response repressor of LexA also had a large negative Δ*w*_Cr_ value (−1.7) indicating that uncontrolled SOS repair under conditions of Cr[VI] stress is detrimental. The SOS-response cell division inhibitor gene *sulA*, also had a large negative Δ*w*_Cr_ (−1.3).

Some of the DNA repair fitness data seen anaerobically with RCH2 in the Cr[VI] challenge mirror what has been seen in other microorganisms under aerobic conditions. For example, in *E. coli*, several SOS genes, including those encoding RecA and a cell division inhibitor, had increased transcription upon Cr[VI] challenge (Llagostera et al., 1986) and in *C. crescentus*, whole-genome transcriptional analysis in response to chromate toxicity revealed upregulation of some of the components of the excision repair system (Hu et al., 2005). In *S. oneidensis* MR-1, Cr[VI] exposure resulted in the upregulation of numerous DNA repair related genes including *recO*, that had a large negative Δ*w*_Cr_ value (−1.9) here but homologs of several upregulated genes in *S. oneidensis* had no significant fitness value change in RCH2 including *uvrD* (0.1) (Chourey et al., 2006).

When grown aerobically in the presence of Cr[VI], many organisms induce production of enzymes known to detoxify ROS, including SOD, catalase (Ackerley et al., 2006), thioredoxin and glutaredoxin (Hu et al., 2005; Chourey et al., 2006). None of the homologs of these genes in RCH2 had significantly negative Δ*w*_Cr_ values when exposed to Cr[VI] anaerobically, including those encoding SOD (0.2), five catalases (0.0–0.3), seven glutaredoxins (0.1–0.2), and five thioredoxins (0.0–0.2). This indicates that the anaerobic approach described herein is an efficient means to deconstruct the direct effects that chromium has on DNA damage from the indirect effects that are mediated by ROS when Cr[VI] exposure occurs in the presence of O_2_.

### Chromate toxicity involving nitrate reduction and Mo cofactor biosynthesis

In the present study, the RB-TNSeq library of RCH2 was challenged with Cr[VI] and U[VI] while growing under denitrifying conditions. As a consequence, genes encoding nitrate reductase and other accessory proteins required for nitrate reductase synthesis and function were expected to be important for fitness in RCH2 (Vaccaro et al., 2016a,b). This was confirmed as in the absence of either Cr or U (control) the nitrate reductase structural genes *narGHI* (*psest_3483, psest_3485*, and *psest_3486*) had *w*_Cont_ ranging from −1.8 to −2.4 (Tables 1, 2). In addition, genes encoding proteins involved in synthesis of the Mo-cofactor (Mo-co), a required component of the catalytic site in nitrate reductase, were also expected to have negative *w*_Cont_, and this proved to be the case. The two Mo-co genes (*psest_3479* and *psest_3480*) that are part of the nitrate reductase operon and encode MoeA and MoaB had *w*_*Cont*_ of −2.1 and −1.6 respectively. Similarly, two unlinked Mo-co genes that encode MoeB and MobA (*psest_1115* and *psest_1961*) had *w*_*Cont*_ values of −2.6 and −2.4 respectively.

**Figure 1:**
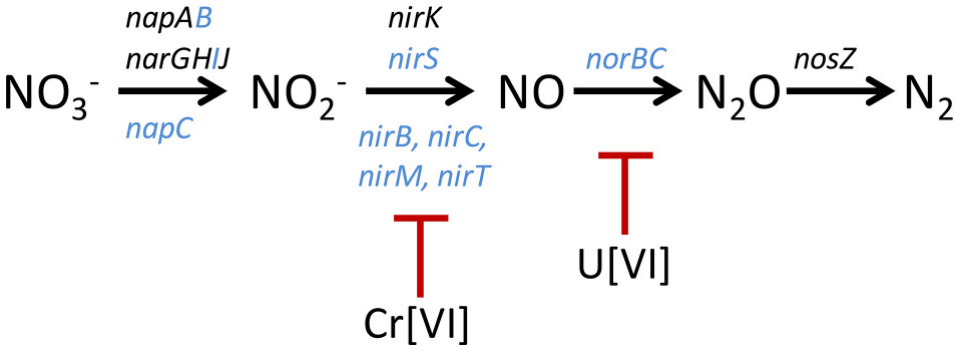
Model of interactions between the denitrification pathway and Cr[VI] and U[VI]. The denitrification pathway is displayed in black with genes encoding structural proteins located above the pathway, and other cytochrome accessory proteins involved in each step located below the pathway. All genes that encode cytochrome proteins are colored blue (Zumft, 1997). Cr[VI] is shown inhibiting the denitrification pathway at the step of nitrite reduction as evidenced by whole cell assays, and U[VI] is hypothesized to inhibit at the step of nitric oxide reduction. The structural genes are; periplasmic nitrate reductase (*nap*), nitrate reductase (*nar*), nitrite reductase (*nir*), nitric oxide reductase (*nor*), and nitrous oxide reductase (*nos*).

In the Cr challenge experiments, many genes involved in nitrate reduction had even larger negative fitness values than those that were measured in the absence of Cr (Table 1). These included the nitrate reductase structural genes *narG* (−1.1) and *narH* (−1.0), Mo-co biosynthesis genes moaA (−1.5), moaB (−1.4), moeA (−1.3), and a component of the molybdate ABC transporter (−1.2) (Table 1). These results show that Cr[VI] interferes with RCH2 growth under denitrifying conditions when nitrate reductase activity is decreased or eliminated. We propose that RCH2 strains lacking nitrate reductase activity survive in the library control growth, albeit with lower fitness, by using the rest of the denitrification pathway to respire (Figure 1). In this case, the presence of Cr[VI] could interfere with a component of the denitrification pathway whose action takes place after nitrate is reduced to nitrite. The likely target(s) of Cr[VI] toxicity in the remainder of the denitrification pathway are one or more of the several cytochrome containing enzymes involved, such as NirS (cytochrome *cd*_1_), a nitrite reductase, or NorBC, the cytochrome *b* and *c* subunits of Nor, required for nitric oxide respiration (Figure 1). From a previous study, the reduction of Cr[VI] in RCH2 under anaerobic conditions was shown to require the presence of nitrate, indicating that a component of the denitrification pathway is involved (Han et al., 2010). It has also been observed that other organisms reduce Cr[VI] under anaerobic conditions using cytochrome components of electron transport chains (Mangaiyarkarasi et al., 2011; Joutey et al., 2015). If Cr[VI] is reduced by RCH2 denitrification cytochromes at the expense of their normal activities, this could represent a Cr[VI] toxicity target in RCH2 (Figure 1).

**Figure 2:**
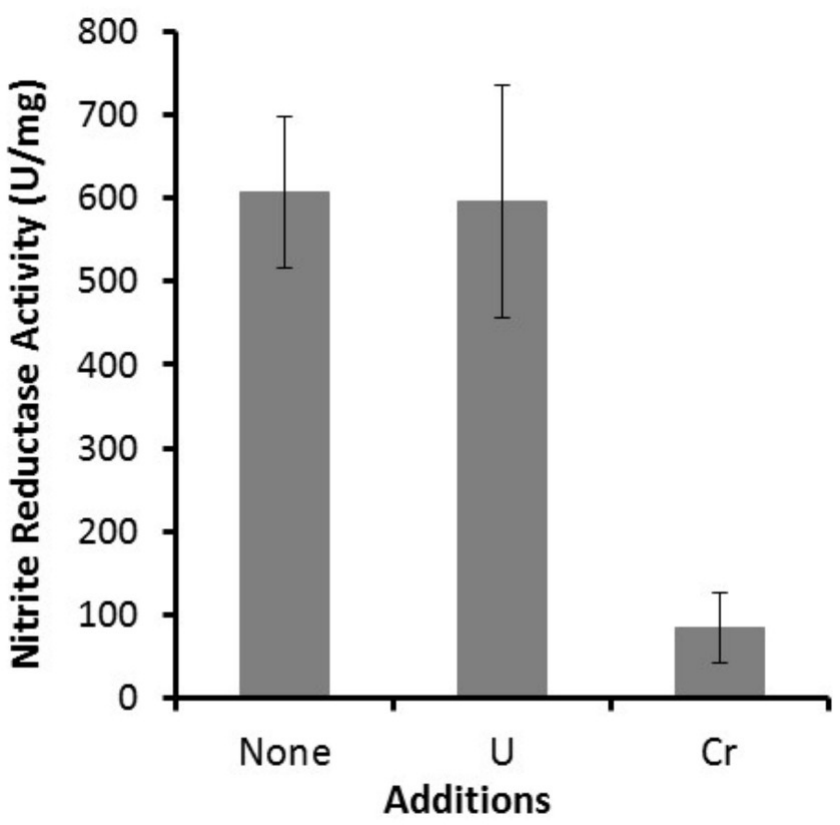
Nitrite reductase activity was measured for whole RCH2 cells. Cells were grown under anaerobic denitrifying conditions with 20 mM furmarate as a carbon source and 20 mM nitrate as an electron acceptor in the presence of no exogenous metal, 3 mM uranyl acetate or 120 μM K_2_Cr_2_O_7_. Nitrite reductase values are reported as Units/mg protein, where a unit corresponds to 1 nmol of nitrite reduced/min.

Nitrite reductase activity was measured for RCH2 whole cells grown under denitrifying conditions in the absence and presence of 120 μM K_2_Cr_2_O_7_ and 3 mM uranyl acetate (Figure 2). This was to test the model that Cr[VI] interferes with cytochrome-containing components of the denitrification pathway downstream of nitrate reductase activity, leading to the large negative Δ*w*_*Cr*_ values seen for nitrate reductase related genes. In support of the model, cells grown in the presence of Cr[VI] had greatly decreased nitrite reductase activity (Figure 2). This indicates that Cr[VI] indeed interferes with nitrite reductase activity, likely through interaction with one or more of the involved cytochrome containing enzymes (NirS, NirB, NirC, NirM, and/or NirT; Figure 1). Many contaminated sites like Hanford, WA, where RCH2 was isolated, and Oak Ridge Reservation, TN are contaminated with both heavy metals like Cr[VI] or U[VI] and nitrate (Riley and Zachara, 1992; Fruchter, 2002). Remediation efforts at sites like these may be complicated by this interaction between metals and the denitrification pathway.

### Chromate toxicity involving sulfur assimilation and intracellular thiols

Chromate is known to interacts with the sulfur assimilation pathway at multiple levels. One of the major ways in which Cr[VI] enters the cell is through the sulfate transporter system (Cervantes et al., 2001) thereby competing with sulfate transport. In addition, sulfide (H_2_S) can directly react with and reduce Cr[VI] to the less mobile and less toxic Cr[III] (Kim et al., 2001). Intracellularly, reduced thiols such as glutathione and ascorbic acid can also reduce Cr[VI] (Arslan et al., 1987; Costa, 2003; Xu et al., 2004). These effects of Cr[VI] on sulfur assimilation were demonstrated in anaerobically-grown RCH2 where the growth defect caused by 80 μM K_2_Cr_2_O_7_ was mitigated by the exogenous addition of various sulfur sources (Figure S1). High concentrations (1 mM) but not low concentrations (0.1 mM) of sulfate partially restored growth, presumably by competing with Cr[VI] uptake through the sulfate transport system. Addition of 100 μM H_2_S also restored growth, presumably by reducing Cr[VI] in the growth medium to the less cell permeable and thus less toxic Cr[III] (Figure S1).

**Figure 3:**
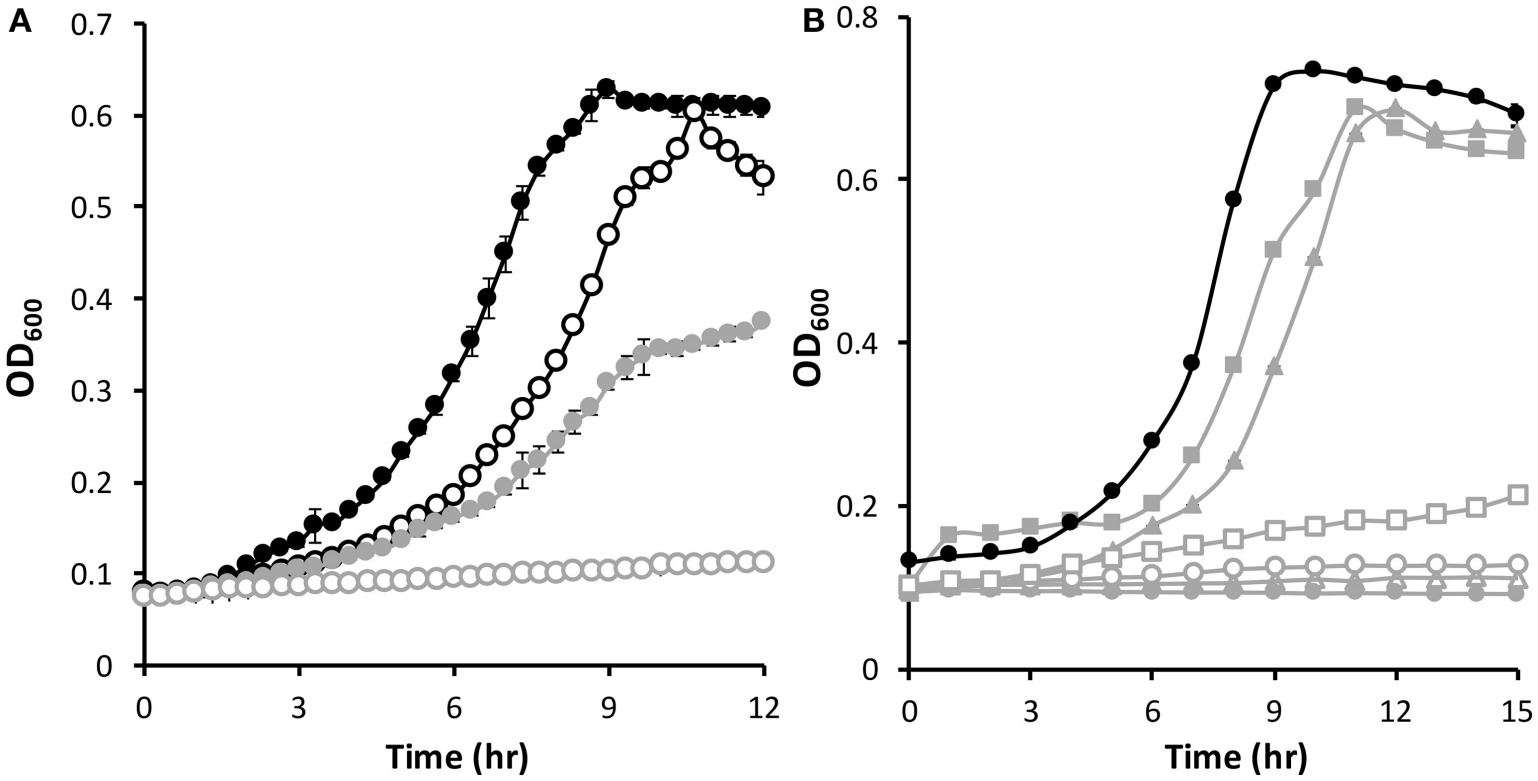
Anaerobic growth of *Pseudomonas stutzeri* RCH2 WT (black) and Δ2088 (gray). **(A)** Growth of WT and Δ2088 in the presence of yeast extract without (filled) and with (open) 25 μM K_2_Cr_2_O_7_ added to the growth medium. **(B)** Growth of WT and Δ2088 without yeast extract (closed circles) and with various exogenously added sulfur sources: 1 mM sulfate (open triangles), 1 mM sulfite (open circles), 0.3 mM sulfide (closed squares), 0.3 mM cysteine (open squares), and 0.1 mM thiosulfate (closed triangles).

One of the largest negative Δ*w*_*Cr*_ values observed during the anaerobic Cr[VI] fitness challenge was for the gene encoding the hypothetical protein Psest_2088 (−2.3), which is located directly downstream of the gene encoding the β subunit of sulfite reductase (*psest_2089*) (no gene fitness data is available for *psest_2089*), and upstream of another gene of unknown function encoded in the opposite direction. Psest_2088 is predicted to be an intracellular protein with a mass of 19.2 kDa and contains a conserved domain of unknown function found in several bacterial proteins (DUF934 superfamily). A deletion mutant lacking *psest_2088* was constructed (Δ2088) and this strain was more sensitive than the wild-type to Cr[VI] (Figure 3A). When grown in the presence of 0.5 g/L yeast extract, the Δ2088 mutant had a growth defect compared to wild-type. However, growth of Δ2088 was severely impaired compaired to wild-type if 25 μM K_2_Cr_2_O_7_ was added to the growth medium (Figure 3A).

**Figure 4:**
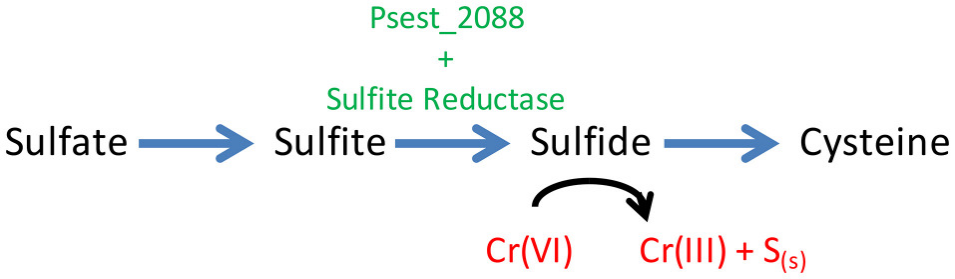
Model of the roles of Psest_2088 and Cr(VI) toxicity in sulfur assimilation. The Psest_2088 protein acts as an accessory protein required for sulfite reductase activity. Also shown is the ability of chromate to oxidize intracellular reduced sulfur pools. The combination of decreased sulfite reductase activity and chromate oxidizing the reduced sulfur pool is responsible for the large fitness defect associated with *psest_2088* grown in the presence of Cr[VI].

Interestingly, the Δ2088 mutant strain is unable to grow in the absence of 0.5 g/L yeast extract unless an additional sulfur source is added to the growth medium. This is similar to how wild-type behaves when Cr[VI] is present (Figure 3B and Figure S1). Amounts of sulfate and sulfite (1 mM) are unable to correct the growth defect, but 0.3 mM cysteine and sulfide partially restore growth (Figure 3B). Thiosulfate (0.1 mM), a sulfur compound that is enzymatically broken down into sulfide and sulfite (Haschke and Campbell, 1971), also restores growth. This growth restoration profile of sulfur sources is consistent with Psest_2088 having an integral role in sulfite reductase activity since sulfur sources after sulfite reductase in the sulfur assimilation pathway restore growth (sulfide and cysteine) where those before (sulfate and sulfite) do not (Figure 4). One possibility is that Psest_2088 is involved in siroheme biosynthesis, a cofactor required by sulfite reductase. These growth phenotypes of Δ2088 were also seen when the cells were grown aerobically (Figure S2), indicating that the function of Psest_2088 is required both under aerobic and anaerobic growth conditions. Cysteine did not restore growth as well as sulfide likely due to a deleterious effect that cysteine has on RCH2 growth in general.

Taken together, these data support a model in which Cr[VI] toxicity is caused at least in part by oxidation of reduced sulfur compounds in the cell or in the oxidation of cellular components that require a reduced sulfur species for function (Figure 4). Strains lacking Psest_2088 have a decreased capacity to reduce sulfite to sulfide and therefore would be more sensitive to Cr[VI] oxidation of intracellular reduced sulfur species. Extracts of RCH2 wild-type cells grown anaerobically in the presence and absence of 50 μM K_2_Cr_2_O_7_ and of Δ2088 were prepared anaerobically and were measured for reduced thiol concentrations. As shown in Figure S3, all of the extracts had comparable concentrations of reduced thiol groups. Although, on the surface the model would predict that the wild-type extract (no Cr[VI] added) should have higher concentrations of reduced thiols than the other extracts, this result is still consistent with the proposed model (Figure 4). RCH2 may strictly maintain a minimum intracellular concentration of reduced thiols during growth and oxidation of thiols by Cr[VI] may result in a slower growth rate rather than in an oxidized intracellular environment.

In addition to *psest_2088*, a negative Δ*w*_*Cr*_ value was exhibited by *psest_4063* (−1.0), the gene encoding the ATP-binding subunit of the sulfate ABC transporter. The other two genes encoding components of the sulfate transporter *psest_4061* (−0.6) and *psest_4062* (−0.8) also had negative Δ*w*_*Cr*_ values. If Cr[VI] enters the cell by multiple transporters and depletes the reduced thiol pool, deletion of a sulfate transporter could have the deleterious effect of decreasing sulfate uptake, even though it may also decrease Cr[VI] uptake. In addition, a gene (*psest_0494*) annotated as a rhodanese-related sulfurtransferase, putatively involved in the transfer of sulfur containing groups, had a large negative Δ*w*_*Cr*_ (−2.1). All three genes of the methionine import system, which could provide a source of reduced sulfur, display large negative Δ*w*_*Cr*_, *psest_4314-4316* (−2.0, −1.5, and −3.0). From the combined data, we propose that the reduced sulfur pool of RCH2 is a target of chromate toxicity (Figure 4).

### Chromate toxicity involving chromate reduction

In our study, large negative Δ*w*_*Cr*_ values were seen for the genes of both cytochrome *cd*_1_ nitrite reductase NirS (−1.6), and for NirF (−1.5), a protein needed for NirS maturation (Nicke et al., 2013). If Cr[VI] interferes with nitrate reduction, nitrite reduction, requiring NirS, would become important for respiration and survival. Anaerobic Cr[VI] reduction has been associated with electron transport systems in many organisms with electrons being transferred to Cr[VI] by various cytochromes (Mangaiyarkarasi et al., 2011; Joutey et al., 2015). For example, *Desulfovibrio vulgaris* soluble cytochrome *c*_3_ was shown to be involved in Cr[VI] reduction (Lovley and Phillips, 1994). In a previous study, the reduction rates of Cr[VI] were measured for cell suspensions of RCH2 under both aerobic and anaerobic conditions (Han et al., 2010). In both cases, similar Cr[VI] reduction rates (4–5 × 10^−12^ μmol. h^−1^. cell^−1^) were observed, however, the presence of O_2_ (in the case of aerobic conditions) or nitrate (in the case of anaerobic conditions) was required for activity in the assay. Cr[VI] reductase activity was not inducible by adding Cr[VI] to the growth medium, and it was concluded that RCH2 could not reduce Cr[VI] unless reducing equivalents for Cr[VI] are first generated in the presence of the physiological electron acceptor (Han et al., 2010).

### Chromate toxicity involving chromate efflux

The Cr[VI] efflux protein ChrA has been shown to confer Cr[VI] resistance to *P. aeruginosa* and other microorganisms (Aguilera et al., 2004; Ramírez-Díaz et al., 2008). RCH2 contains three homologs of ChrA, Psest_1915 and two proteins that are identical in sequence, Psest_0945 and Psest_4025. There was not a negative Δ*w*_*Cr*_ value for *psest_1915* (0.1) and the other two ChrA homologs do not have gene fitness values as no transposon insertions were present in these genes in the RB-TNSeq library. Hence, without further information, the role of efflux in RCH2 Cr[VI] resistance cannot be determined.

### Chromate toxicity involving hypothetical proteins

There were ten genes of unknown function with large negative Δ*w*_*Cr*_ values ranging from −1.0 to −1.6 (Table 1). The function of these proteins in Cr[VI] resistance could be the basis of a future endeavor. Tools such as RB-TNSeq can be used to discover multiple fitness defects for genes of unknown function under diverse conditions, elucidating the roles these proteins have in cellular metabolism.

### Uranyl toxicity involving nitrate reduction and Mo-cofactor biosynthesis

In the U challenge experiments, as with the Cr[VI] challenge, genes related to nitrate reduction had large negative Δ*w*_*U*_ values (Table 2). These large negative Δ*w*_U_ values were seen for the denitrification required nitrate reductase structural genes *narGHI* (−1.4 to −1.8), Mo-co biosynthesis genes *moaB* (−3.5), *moeA* (−2.2), *mobA* (−1.7), *moaC* (−1.3), and two genes of the molybdate ABC transporter (−1.1 and −1.2). In general, the Δ*w*_*U*_ for nitrate reduction related genes were more pronounced and widespread than the Δ*w*_*Cr*_. This may simply indicate that the U[VI] challenge was more effective than the Cr[VI] challenge, or uranium toxicity may have a greater effect on this area of metabolism. We propose a similar model for the effect of U[VI] on nitrate reduction gene fitness values as we did for Cr[VI], in that U[VI] likely interacts with cytochromes required for the steps in the denitrification pathway after nitrate is reduced to nitrite (Figure 1). U[VI] reduction by bacteria is not catalyzed by specialized reductases but by redox enzymes that normally function in other cellular processes (Wall and Krumholz, 2006). These include cytochromes such as cytochrome *c3* from *D. vulgaris* (Payne et al., 2002). If U[VI] is reduced by or interacts with one or more of the denitrification cytochromes of RCH2, this could disrupt the later steps of denitrification explaining the large negative Δ*w*_U_ seen for genes involved in nitrate reduction. Althought U[VI] does not decrease RCH2 whole cell nitrite reductase activity (Figure 2), U[VI] may still interfere with the cytochrome containing nitric oxide reductase resulting in the large negative Δ*w*_*U*_ observed for nitrate reductase related genes.

### Uranyl toxicity involving stress proteins

A large negative Δ*w*_*U*_ value was observed for *psest_3488* (−1.9), which encodes the universal stress protein UspA. Expression of the *uspA* gene has been shown to be up-regulated under several stress conditions in *E. coli* including starvation, heat, oxidants, metals, uncouplers, ethanol and antibiotics (Kvint et al., 2003). This stress protein also appears to play a role in uranium resistance in RCH2. In *E. coli* oxidative stress genes including SOD and catalase were also connected with resistance to uranium toxicity under aerobic conditions (Khemiri et al., 2014). In our RCH2 experiment, the gene for SOD and the five annotated catalase genes did not have large negative Δ*w*_*U*_ (0.0–0.2), presumably due to the anaerobic growth conditions used in the experiment.

### Uranyl toxicity involving exopolysaccharide synthesis

Two genes involved in the formation of polysaccharides had large negative Δ*w*_*U*_ values, *psest_2232* (−4.3) and *psest_0993* (−2.3). They encode UTP-glucose-1-phosphate uridylyltransferase and glucose-6-phosphate isomerase, respectively. These enzymes are involved in the synthesis of UDP-glucose, which is a building block needed to form the polysaccharide glycogen (Alonso et al., 1995). We propose that RCH2 produces an exopolysaccharide using both Psest_2232 and Psest_0993 that is important for uranium resistance. Previously *Pseudomonas* sp. EPS-5028 and *Acidithiobacillus ferrooxidans* were shown to accumulate uranium on exopolysaccharides under aerobic conditions (Marqués et al., 1990; Merroun et al., 2003), but it was not demonstrated that this accumulation prevented U toxicity. The data presented here supports the idea that under anaerobic conditions, accumulation of uranium on exopolysaccaride constitutes a defense mechanism.

### Uranyl toxicity involving hypothetical proteins

There were five hypothetical proteins that had large negative Δ*w*_*U*_ values ranging from −1.0 to −1.5 (Table 2). The function of these proteins in uranium resistance is not known and these will be the basis of future research.

## Conclusions

Many new insights were gained through the course of this genome-wide fitness analysis on the targets of Cr[VI] and U[VI] toxicity in RCH2 grown under denitrifying conditions, as well as the defense mechanisms RCH2 uses to defend itself against these metals. For Cr[VI], DNA is a toxicity target even under anaerobic conditions. Cr[VI]-dependent fitness defects were seen under anaerobic conditions for strains lacking proteins involved in homologous recombination and nucleotide excision DNA repair. This is a result of direct DNA damage by Cr in the absence of O_2_, as the generation of DNA damaging ROS is another route of Cr toxicity observed in aerobic experiments (Arslan et al., 1987; Costa, 2003; Xu et al., 2004). Fitness data together with physiological growth studies on wild-type RCH2 and the Δ2088 mutant strain were used to develop a model in which the reduced thiol pool is an additional target of Cr[VI] toxicity (Figure 4). In this model, Psest_2088, a protein of previously unknown function, is a key protein involved in sulfur assimilation at the step of sulfite reduction. Both Cr[VI] and U[VI] toxicity have large fitness effects on RCH2 strains with defects in nitrate reduction. We propose that both metals interfere with cytochrome components of the remainder of the denitrification pathway, which is critical to respiration and survival when nitrate reduction is hindered (Figure 1). This could hinder the remediation of sites contaminated with both nitrate and heavy metals such as Cr[VI] and U[VI]. Finally, exopolysaccharide biosynthesis and the universal stress protein UspA were identified as possible defenses mechanisms against U[VI] toxicity. Cr[VI] and U[VI] damage living organisms in diverse ways, and RB-TnSeq technology is a powerful tool that can be used to study these processes, and identify the metabolic pathways involved.

## Originality-significance statement

Much of what has been observed regarding chromium (Cr[VI]) and uranium (U[VI]) toxicity has been studied under aerobic conditions in which metal toxicity is caused not only directly by the metal, but also indirectly due to redox reactions of the metal with oxygen and the resulting reactive oxygen species. Herein we report the results of random barcode transposon site sequencing experiments performed on the chromium-contaminated environmental isolate, *Pseudomonas stutzeri* RCH2, grown under anaerobic denitrifying conditions. This combined with other techniques has allowed us to elucidate on a genome wide scale the anaerobic toxicity targets of Cr[VI] and U[VI] and the mechanisms used by RCH2 to defend against these metals.

## Author contributions

MT, WL, and XG designed and performed the experiments, analyzed and interpreted the data. GZ and AY constructed deletion mutant strains. KW carried out the DNA sequencing and provided support for the RB-TNSeq experiments. MT wrote the manuscript and BV, FP, AD, AA, JW, and MA contributed input and critically reviewed the manuscript. MA supervised the work.

### Conflict of interest statement

The authors declare that the research was conducted in the absence of any commercial or financial relationships that could be construed as a potential conflict of interest.

## References

[B1] Ackerley D. Barak Y. Lynch S. Curtin J. Matin A. (2006). Effect of chromate stress on *Escherichia coli* K-12. J. Bacteriol. 188, 3371–3381. 10.1128/JB.188.9.3371-3381.2006 16621832 PMC1447458

[B2] Aguilera S. Aguilar M. E. Chávez M. P. López-Meza J. E. Pedraza-Reyes M. Campos-García J.. (2004). Essential residues in the chromate transporter ChrA of *Pseudomonas aeruginosa*. FEMS Microbiol. Lett. 232, 107–112. 10.1016/S0378-1097(04)00068-0 15019742

[B3] Alonso M. Lomako J. Lomako W. Whelan W. (1995). A new look at the biogenesis of glycogen. FASEB J. 9, 1126–1137. 7672505 10.1096/fasebj.9.12.7672505

[B4] Alvarez A. H. Moreno-Sánchez R. Cervantes C. (1999). Chromate efflux by means of the ChrA chromate resistance protein from *Pseudomonas aeruginosa*. J. Bacteriol. 181, 7398–7400. 10572148 10.1128/jb.181.23.7398-7400.1999 PMC103707

[B5] Arslan P. Beltrame M. Tomasi A. (1987). Intracellular chromium reduction. BBA Mol. Cell Res. 931, 10–15. 10.1016/0167-4889(87)90044-9 2820507

[B6] Ayres R. U. (1992). Toxic heavy metals: materials cycle optimization. Proc. Natl. Acad. Sci. U.S.A. 89, 815–820. 10.1073/pnas.89.3.815 11607259 PMC48332

[B7] Beneš P. (1999). The environmental impacts of uranium mining and milling and the methods of their reduction, in Chemical Separation Technologies and Related Methods of Nuclear Waste Management. NATO Science Series (Series 2: Environmental Security), eds Choppin G. R. Khankhasayev M. K. (Dordrecht: Springer).

[B8] Bradford M. M. (1976). A rapid and sensitive method for the quantitation of microgram quantities of protein utilizing the principle of protein-dye binding. Anal. Biochem. 72, 248–254. 10.1016/0003-2697(76)90527-3 942051

[B9] Cervantes C. Campos-García J. Devars S. Gutiérrez-Corona F. Loza-Tavera H. Torres-Guzmán J. C.. (2001). Interactions of chromium with microorganisms and plants. FEMS Microbiol. Rev. 25, 335–347. 10.1111/j.1574-6976.2001.tb00581.x 11348688

[B10] Cervantes-Cervantes M. Hadjeb N. Newman L. A. Price C. A. (1990). ChrA is a carotenoid-binding protein in chromoplasts of Capsicum annuum. Plant Physiol. 92, 1241–1243. 10.1104/pp.92.4.1241 16667396 PMC1062442

[B11] Cheung K. Gu J.-D. (2007). Mechanism of hexavalent chromium detoxification by microorganisms and bioremediation application potential: a review. Int. Biodeter. Biodegr. 59, 8–15. 10.1016/j.ibiod.2006.05.002

[B12] Chourey K. Thompson M. R. Morrell-Falvey J. Verberkmoes N. C. Brown S. D. Shah M.. (2006). Global molecular and morphological effects of 24-hour chromium (VI) exposure on *Shewanella oneidensis* MR-1. Appl. Environ. Microb. 72, 6331–6344. 10.1128/AEM.00813-06 16957260 PMC1563591

[B13] Costa M. (2003). Potential hazards of hexavalent chromate in our drinking water. Toxicol. Appl. Pharm. 188, 1–5. 10.1016/S0041-008X(03)00011-5 12668116

[B14] Czakó-Vér K. Batiè M. Raspor P. Sipiczki M. Pesti M. (1999). Hexavalent chromium uptake by sensitive and tolerant mutants of Schizosaccharomyces pombe. FEMS Microbiol. Lett. 178, 109–115. 10.1016/S0378-1097(99)00342-0 10483729

[B15] Dayan A. Paine A. (2001). Mechanisms of chromium toxicity, carcinogenicity and allergenicity: review of the literature from 1985 to 2000. Hum. Exp. Toxicol. 20, 439–451. 10.1191/096032701682693062 11776406

[B16] Fruchter J. (2002). Peer reviewed: In-situ treatment of chromium-contaminated groundwater. Environ. Sci. Technol. 36, 464A–472A. 10.1021/es022466i 12523403

[B17] Grevatt P. C. (1998). Toxicological Review of Hexavalent Chromium. Support of Summary Information on the Integrated Risk Information System (IRIS), US Environmental Protection Agency Washington DC.

[B18] Gupta V. Agarwal S. Saleh T. A. (2011). Chromium removal by combining the magnetic properties of iron oxide with adsorption properties of carbon nanotubes. Water Res. 45, 2207–2212. 10.1016/j.watres.2011.01.012 21303713

[B19] Han R. Geller J. T. Yang L. Brodie E. L. Chakraborty R. Larsen J. T.. (2010). Physiological and transcriptional studies of Cr (VI) reduction under aerobic and denitrifying conditions by an aquifer-derived pseudomonad. Environ. Sci. Technol. 44, 7491–7497. 10.1021/es101152r 20822129

[B20] Haschke R. H. Campbell L. L. (1971). Thiosulfate reductase of Desulfovibrio vulgaris. J. Bacteriol. 106, 603–607. 10.1128/jb.106.2.603-607.1971 PMC285136 5573735

[B21] Hu P. Brodie E. L. Suzuki Y. Mcadams H. H. Andersen G. L. (2005). Whole-genome transcriptional analysis of heavy metal stresses in Caulobacter crescentus. J. Bacteriol. 187, 8437–8449. 10.1128/JB.187.24.8437-8449.2005 16321948 PMC1317002

[B22] Joutey N. T. Sayel H. Bahafid W. El Ghachtouli N. (2015). Mechanisms of hexavalent chromium resistance and removal by microorganisms, in Reviews of Environmental Contamination and Toxicology Volume 233. Reviews of Environmental Contamination and Toxicology (Continuation of Residue Reviews), ed Whitacre D. (Cham: Springer). 10.1007/978-3-319-10479-9_2 25367133

[B23] Khemiri A. Carrière M. Bremond N. Mlouka M. A. B. Coquet L. Llorens I.. (2014). *Escherichia coli* response to uranyl exposure at low pH and associated protein regulations. PLoS ONE 9:e89863. 10.1371/journal.pone.0089863 24587082 PMC3935937

[B24] Kim C. Zhou Q. Deng B. Thornton E. C. Xu H. (2001). Chromium (VI) reduction by hydrogen sulfide in aqueous media: stoichiometry and kinetics. Environ. Sci. Technol. 35, 2219–2225. 10.1021/es0017007 11414022

[B25] Kortenkamp A. O'brien P. (1994). The generation of DNA single-strand breaks during the reduction of chromate by ascorbic acid and/or glutathione *in vitro*. Environ. Health Persp. 102:237 10.1289/ehp.94102s3237 PMC1567429 7843105

[B26] Kvint K. Nachin L. Diez A. Nyström T. (2003). The bacterial universal stress protein: function and regulation. Curr. Opin. Microbiol. 6, 140–145. 10.1016/S1369-5274(03)00025-0 12732303

[B27] Llagostera M. Garrido S. Guerrero R. Barbé J. (1986). Induction of SOS genes of *Escherichia coli* by chromium compounds. Environ. Mutagen. 8, 571–577. 10.1002/em.2860080408 3525136

[B28] Lovley D. R. Phillips E. J. (1994). Reduction of chromate by Desulfovibrio vulgaris and its c3 cytochrome. Appl. Environ. Microb. 60, 726–728. 10.1128/aem.60.2.726-728.1994 PMC201373 16349200

[B29] Lovley D. R. Widman P. K. Woodward J. C. Phillips E. (1993). Reduction of uranium by cytochrome c3 of Desulfovibrio vulgaris. Appl. Environ. Microb. 59, 3572–3576. 8285665 10.1128/aem.59.11.3572-3576.1993 PMC182500

[B30] Mangaiyarkarasi M. M. Vincent S. Janarthanan S. Rao T. S. Tata B. (2011). Bioreduction of Cr (VI) by alkaliphilic Bacillus subtilis and interaction of the membrane groups. Saudi J. Biol. Sci. 18, 157–167. 10.1016/j.sjbs.2010.12.003 23961119 PMC3730869

[B31] Marqués A. M. Bonet R. Simon-Pujol M. D. Fusté M. C. Congregado F. (1990). Removal of uranium by an exopolysaccharide from *Pseudomonas* sp. Appl. Environ. Microb. 34, 429–431.

[B32] Martins M. Faleiro M. L. Da Costa A. M. R. Chaves S. Tenreiro R. Matos A. P.. (2010). Mechanism of uranium (VI) removal by two anaerobic bacterial communities. J. Hazard Mater. 184, 89–96. 10.1016/j.jhazmat.2010.08.009 20832165

[B33] Merroun M. L. Nedelkova M. Ojeda J. J. Reitz T. Fernández M. L. Arias J. M.. (2011). Bio-precipitation of uranium by two bacterial isolates recovered from extreme environments as estimated by potentiometric titration, TEM and X-ray absorption spectroscopic analyses. J. Hazard Mater. 197, 1–10. 10.1016/j.jhazmat.2011.09.049 22019055

[B34] Merroun M. L. Raff J. Rossberg A. Hennig C. Reich T. Selenska-Pobell S. (2005). Complexation of uranium by cells and S-layer sheets of *Bacillus sphaericus* JG-A12. Appl. Environ. Microb. 71, 5532–5543. 10.1128/AEM.71.9.5532-5543.2005 16151146 PMC1214696

[B35] Merroun M. Hennig C. Rossberg A. Reich T. Selenska-Pobell S. (2003). Characterization of U (VI)-*Acidithiobacillus ferrooxidans* complexes using EXAFS, transmission electron microscopy, and energy-dispersive X-ray analysis. Radiochim. Acta 91, 583–592. 10.1524/ract.91.10.583.22477

[B36] Morimatsu K. Kowalczykowski S. C. (2003). RecFOR proteins load RecA protein onto gapped DNA to accelerate DNA strand exchange: a universal step of recombinational repair. Mol. Cell 11, 1337–1347. 10.1016/S1097-2765(03)00188-6 12769856

[B37] Nicke T. Schnitzer T. Münch K. Adamczack J. Haufschildt K. Buchmeier S.. (2013). Maturation of the cytochrome cd1 nitrite reductase NirS from *Pseudomonas aeruginosa* requires transient interactions between the three proteins NirS, NirN and NirF. Bioscience Rep. 33:e00048. 10.1042/BSR20130043 23683062 PMC3694632

[B38] Nies A. Nies D. H. Silver S. (1990). Nucleotide sequence and expression of a plasmid-encoded chromate resistance determinant from *Alcaligenes eutrophus*. J. Biol. Chem. 265, 5648–5653. 2180932

[B39] Nishioka H. (1975). Mutagenic activities of metal compounds in bacteria. Mutat. Res.-Envir. Muta. 31, 185–189. 10.1016/0165-1161(75)90088-6 805366

[B40] Ohtake H. I. S. A. O. Silver S. (1994). Bacterial detoxification of toxic chromate, in Biological Degradation and Bioremediation of Toxic Chemicals (London: Chapman & Hall), 403–415.

[B41] Payne R. B. Gentry D. M. Rapp-Giles B. J. Casalot L. Wall J. D. (2002). Uranium reduction by Desulfovibrio desulfuricans strain G20 and a cytochrome c3 mutant. Appl. Environ. Microb. 68, 3129–3132. 10.1128/AEM.68.6.3129-3132.2002 12039777 PMC123926

[B42] Petrilli F. L. De Flora S. (1977). Toxicity and mutagenicity of hexavalent chromium on *Salmonella typhimurium*. Appl. Environ. Microb. 33, 805–809. 326184 10.1128/aem.33.4.805-809.1977 PMC170770

[B43] Pimentel B. E. Moreno-Sánchez R. Cervantes C. (2002). Efflux of chromate by *Pseudomonas aeruginosa* cells expressing the ChrA protein. FEMS Microbiol. Lett. 212, 249–254. 10.1111/j.1574-6968.2002.tb11274.x 12113942

[B44] Ramírez-Díaz M. I. Díaz-Pérez C. Vargas E. Riveros-Rosas H. Campos-García J. Cervantes C. (2008). Mechanisms of bacterial resistance to chromium compounds. Biometals 21, 321–332. 10.1007/s10534-007-9121-8 17934697

[B45] Riley R. G. Zachara J. (1992). Chemical *Contaminants* on DOE *L*ands and *Selection* of *Contaminant Mixtures* for *Subsurface Science Research*. Richland, WA: Pacific Northwest Lab.

[B46] Sedlak J. Lindsay R. H. (1968). Estimation of total, protein-bound, and nonprotein sulfhydryl groups in tissue with Ellman's reagent. Anal. Biochem. 25, 192–205. 10.1016/0003-2697(68)90092-4 4973948

[B47] Thatoi H. Das S. Mishra J. Rath B. P. Das N. (2014). Bacterial chromate reductase, a potential enzyme for bioremediation of hexavalent chromium: a review. J. Environ. Manage. 146, 383–399. 10.1016/j.jenvman.2014.07.014 25199606

[B48] Thorgersen M. P. Adams M. W. (2016). Nitrite reduction assay for whole pseudomonas cells. Bio Protocol 6:e1818 10.21769/BioProtoc.1818

[B49] Thorgersen M. P. Lancaster W. A. Rajeev L. Ge X. Vaccaro B. J. Poole F. L.. (2016). A highly expressed high molecular weight S-Layer complex of pelosinus strain UFO1 binds Uranium. Appl. Environ. Microb. 83, 3016–3044. 10.1128/AEM.03044-16 27913415 PMC5288816

[B50] Vaccaro B. J. Lancaster W. A. Thorgersen M. P. Zane G. M. Younkin A. D. Kazakov A. E.. (2016a). Novel metal cation resistance systems from mutant fitness analysis of denitrifying *Pseudomonas stutzeri*. Appl. Environ. Microb. 82, 6046–6056. 10.1128/AEM.01845-16 27474723 PMC5038046

[B51] Vaccaro B. J. Thorgersen M. P. Lancaster W. A. Price M. N. Wetmore K. M. Poole F. L.. (2016b). Determining roles of accessory genes in denitrification by mutant fitness analyses. Appl. Environ. Microb. 82, 51–61. 10.1128/AEM.02602-15 26452555 PMC4702625

[B52] Venitt S. Levy L. (1974). Mutagenicity of chromates in bacteria and its relevance to chromate carcinogenesis. Nature 250, 493–495. 10.1038/250493a0 4620019

[B53] Wall J. D. Krumholz L. R. (2006). Uranium reduction. Annu. Rev. Microbiol. 60, 149–166. 10.1146/annurev.micro.59.030804.121357 16704344

[B54] Wetmore K. M. Price M. N. Waters R. J. Lamson J. S. He J. Hoover C. A.. (2015). Rapid quantification of mutant fitness in diverse bacteria by sequencing randomly bar-coded transposons. MBio 6, e00306–e00315. 10.1128/mBio.00306-15 25968644 PMC4436071

[B55] Widdel F. Bak F. (1992). Gram-negative mesophilic sulfate-reducing bacteria, in The Prokaryotes, eds Balows A. Trüper H. G. Dworkin M. Harder W. Schleifer K. H. (New York, NY: Springer).

[B56] Xu X.-R. Li H.-B. Li X.-Y. Gu J.-D. (2004). Reduction of hexavalent chromium by ascorbic acid in aqueous solutions. Chemosphere 57, 609–613. 10.1016/j.chemosphere.2004.07.031 15488923

[B57] Zumft W. G. (1997). Cell biology and molecular basis of denitrification. Microbiol. Mol. Biol. R. 61, 533–616. 9409151 10.1128/mmbr.61.4.533-616.1997 PMC232623

