# Supplementary material for: Mechanisms of Chromium and Uranium Toxicity in Pseudomonas stutzeri RCH2 Grown under Anaerobic Nitrate-Reducing Conditions
Source: Front Microbiol. 2017 Aug 10;8:1529. doi: 10.3389/fmicb.2017.01529 (PMC5554334; doi:10.3389/fmicb.2017.01529)
Supplement: Supplementary file 1 [file Presentation1.pdf]

Frontiers in Microbiology

Mechanisms of Uranium and Chromium Toxicity in *Pseudomonas stutzeri* RCH2 Grown Under Anaerobic Nitrate-Reducing Conditions

Michael P. Thorgersen[a], W. Andrew Lancaster[a], Xiaoxuan Ge[a], Grant M. Zane[b], Kelly M. Wetmore[c], Brian J. Vaccaro[a], Farris L. Poole[a], Adam Younkin[b], Adam M. Deutschbauer[c], Adam P. Arkin[c], Judy D. Wall[b] and Michael W. W. Adams[a#]

[a]Department of Biochemistry & Molecular Biology, University of Georgia, Athens, GA, 30602

[b]Department of Biochemistry, University of Missouri, Columbia, Mo 65211

[c]Environmental Genomics and Systems Biology Division, Lawrence Berkeley National Laboratory, Berkeley, CA 94720

Running Title: Anaerobic U and Cr Toxicity

Supplementary Material

Tables S1 – S4

Figures S1 – S3

Table S1. Quality metrics for RB-TNSeq data

Condition	Median SE	SE Min	SE Max	MAD12	Average Reads/Gene
Control	0.148	0.002	1.7	0.26	1494
120 µM $K_2Cr_2O_7$	0.154	0.003	1.6	0.27	1378
3 mM Uranyl Acetate	0.152	0.002	2.0	0.26	1475

Standard error (SE) was calculated for each gene under all three conditions. The MAD12 is the median absolute difference {Wetmore, 2015}. The number of reads for each gene from all sequencing runs within a condition were summed and the average value is reported (Average Reads/Gene).

Table S2. Table of primers used for construction of marker-exchange plasmid in this study.

Primer Name	Primer Sequence*
Psest_2088-upF	**CGGCTGCAACTTTGTCATGCTTG**CGCTATAGCTTCGGTGAGGTGC
Psest_2088-upR	**TCGCCTTCTTGACGAGTTCTTCTGA**CGTTCTTAATGATTCGCTGCATAGACGC
Psest_2088-dnF	**GCGCCCCAGCTGGCAATTCCGG**GCTTCATTGATACATCCAGAAATTAGGGTGTTCG
Psest_2088-dnR	**CGAGGCATTTCTGTCCTGGCTGG**GGTACTCATTTCCAGGCTAGCGTTCTG
SpecRpUC-F	CCAGCCAGGACAGAAATGCCTCG
SpUrT-R	CAAGCATGACAAAGTTGCAGCCG
Kan gene Prom Nterm	CCGGAATTGCCAGCTGGGGCGC
KanR	TCAGAAGAACTCGTCAAGAAGGCGA
oriT-out	CCTCGTTCAACAGGTCCAGGG
pMO719-XbaI-dn	TGGGTTCGTGCCTTCATCCG
Km_int_Fwd_revcomp	CTCATCCTGTCTCTTGATCAGATCT
Kan_out_C	CTGACCGCTTCCTCGTGC

* Bolded region of primers are overhang used for cloning with plasmid backbone or kanamycin-resistance marker.

Table S3. Genes with Δ*wCr* ≥ 1

		w_{ctrl}	w_{ctrl}	*wCr*	*wCr*	Δ*wCr*
Locus Tag	Gene Function	AVE	STDEV	AVE	STDEV	Delta
**DNA Repair**						
Psest_3100	RNA polymerase sigma factor RpoE	0.3	0.1	1.3	0.2	1.0
**Sulfur Assimilation**						
Psest_0186	Protein-disulfide isomerase	-0.5	0.4	0.4	0.3	1.0
**Fe Related**						
Psest_0954	Fe-S oxidoreductase	-2.2	0.3	0.4	0.1	2.6
Psest_0953	Iron-sulfur cluster-binding protein	-2.2	0.2	0.2	0.3	2.4
Psest_1463	Fe-S center assembly, SufE	-0.6	1.0	0.5	0.6	1.2
**Hypothetical**						
Psest_3489	Hypothetical protein	-1.3	0.0	1.9	0.2	3.2
Psest_0952	Uncharacterized conserved protein	-2.7	0.5	0.2	0.5	2.8
Psest_4003	TIGR00153 family protein	-1.4	0.5	0.6	0.4	2.1
Psest_2385	Hypothetical protein	-0.6	0.7	0.5	1.2	1.1
**Other**						
Psest_3490	Signal transduction histidine kinase	-1.2	0.9	1.5	0.6	2.7
Psest_0999	Response regulator	-0.7	0.5	1.0	0.6	1.7
Psest_0951	FAD/FMN-containing dehydrogenases	-0.6	0.2	1.0	0.1	1.6
Psest_2418	Diguanylate cyclase (GGDEF) domain	-0.7	0.4	0.9	0.4	1.6
Psest_3042	Nitrate reductase cytochrome c-type , NapB	-0.6	0.2	0.8	1.3	1.4
Psest_3573	dTDP-4-dehydrorhamnose reductase	0.0	0.5	1.4	0.3	1.4
Psest_1974	Integration host factor, IhfA	-1.0	0.3	0.4	0.0	1.4
Psest_0092	Phosphonate C-P lyase system protein PhnK	-0.9	0.6	0.5	0.3	1.4
Psest_2590	3-isopropylmalate dehydratase, small subunit	-0.9	0.5	0.4	0.2	1.3
Psest_2988	Aspartate kinase, monofunctional class	-4.0	0.7	-2.7	0.3	1.3
Psest_0722	Predicted ATPase	-0.9	0.4	0.3	0.6	1.2
Psest_0466	Lipopolysaccharide kinase (Kdo/WaaP)	-1.2	0.5	0.0	0.2	1.2
Psest_0839	cAMP-binding proteins	-0.1	0.2	1.1	0.2	1.2
Psest_2023	Isocitrate dehydrogenase	-1.3	1.2	-0.1	0.3	1.2
Psest_3323	Ribonuclease, Rne/Rng family	-0.8	0.2	0.4	0.5	1.2
Psest_3377	FKBP-type prolyl cis-trans isomerases	-0.1	0.9	1.0	1.0	1.1
Psest_3226	Predicted membrane protein	-0.6	0.5	0.5	1.1	1.1
Psest_3684	Di- and tricarboxylate transporters	-0.2	0.2	0.8	0.1	1.1
Psest_0277	Non-canonical purine NTP pyrophosphatase	-0.3	0.6	0.7	0.3	1.0
Psest_0575	HrpA-like helicases	-0.8	0.2	0.2	1.1	1.0
Psest_4287	Predicted glutamine amidotransferases	-0.2	0.2	0.8	0.4	1.0
Psest_2123	Carbohydrate kinase	-0.8	0.4	0.2	0.3	1.0
Psest_1994	Amidases related to nicotinamidase	0.1	0.1	1.1	0.2	1.0
Psest_1724	Anti-anti-sigma regulatory factor	-1.5	0.4	-0.5	0.3	1.0
Psest_2949	Aspartyl aminopeptidase	0.0	0.3	1.0	0.2	1.0

Psest_0800	Dehydrogenases	-0.1	0.1	0.9	0.0	1.0
Psest_3047	Methionyl-tRNA synthetase	-0.2	0.6	0.8	1.0	1.0
Psest_0393	Methylase of chemotaxis methyl-accepting	-2.2	0.4	-1.2	0.2	1.0
Psest_0325	Putative threonine efflux protein	0.2	0.1	1.2	0.1	1.0

Table S4. Genes with Δ*wU* ≥ 1

		w_{ctrl}	w_{ctrl}	wU	wU	ΔwU
Locus Tag	Gene Function	AVE	STDEV	AVE	STDEV	Delta
**Fe Related**						
Psest_0954	Fe-S oxidoreductase	-2.2	0.3	-0.7	0.2	1.5
Psest_1463	Fe-S center assembly, SufE	-0.6	1.0	0.8	1.0	1.4
Psest_0953	Iron-sulfur cluster-binding protein	-2.2	0.2	-0.8	0.3	1.4
**Hypothetical**						
Psest_0952	Uncharacterized conserved protein	-2.7	0.5	-0.8	0.5	1.9
Psest_3146	Hypothetical protein	-0.9	0.4	1.0	0.5	1.8
Psest_1920	Uncharacterized protein conserved in bacteria	-0.9	0.5	0.6	1.1	1.5
**Other**						
Psest_2243	Glyceraldehyde-3-phosphate dehydrogenase	-0.4	0.8	0.7	2.0	1.2
Psest_1082	Enoyl-CoA hydratase/carnithine racemase	-1.5	0.3	-0.4	0.3	1.1

Figure S1

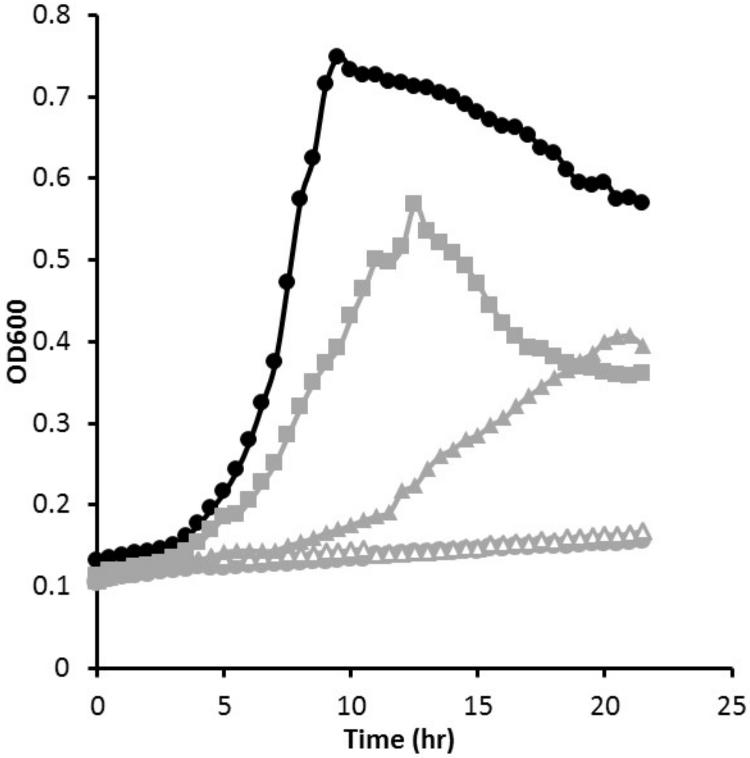

Anaerobic growth of *Pseudomonas stutzeri* RCH2 without (black) and with (gray) the addition of 80 μM $K_2Cr_2O_7$ to the growth medium (not containing yeast extract). Various exogenously added sulfur sources include: none (closed circles), 0.1 mM sulfate (open triangles), 1 mM sulfate (closed triangles), and 0.1 mM sulfide (closed squares).

Figure S2

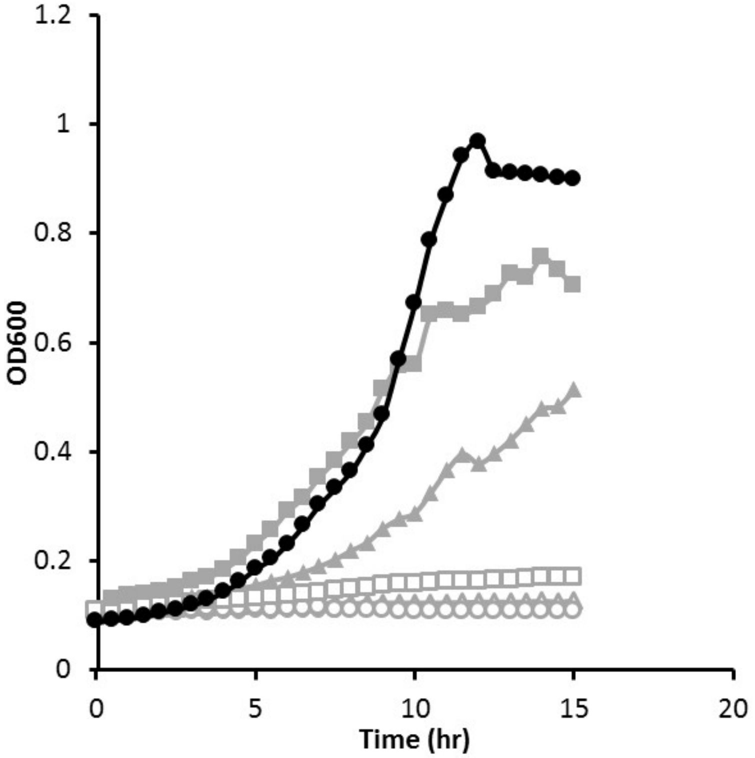

Aerobic growth of *Pseudomonas stutzeri* RCH2 WT (black) and Δ2088 (gray) without yeast extract (closed circles) and with various exogenously added sulfur sources: 1 mM sulfate (open triangles), 1 mM sulfite (open circles), 0.3 mM sulfide (closed squares), 0.3 mM cystein (open squares), and 0.1 mM thiosulfate (closed triangles).

Figure S3

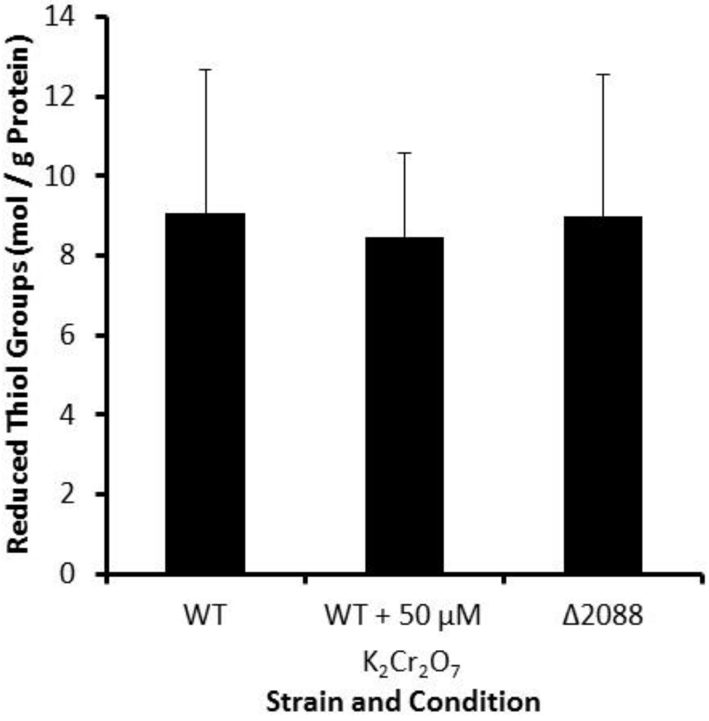

Reduced intracellular thiol concentrations of cell free extracts prepared anaerobically from anaerobically grown cultures of *Pseudomonas stutzeri* RCH2.
